# Enhanced Efficacy of Laser‐Activated Irrigation (Er,Cr:YSGG) in Eradicating *Enterococcus faecalis* Biofilm in 3D‐Printed Molar Replicas: A Pilot Study

**DOI:** 10.1002/cre2.70279

**Published:** 2026-02-19

**Authors:** Margarida Macedo, João Albernaz Neves, Alejandro R. Pérez, Luís Proença, Lucinda J. Bessa

**Affiliations:** ^1^ Egas Moniz School of Health & Science Instituto Universitário Egas Moniz Almada Portugal; ^2^ Department of Endodontics Rey Juan Carlos University Madrid Spain; ^3^ Egas Moniz Research for Interdisciplinary Research (CiiEM) Egas Moniz School of Health & Science Almada Portugal

**Keywords:** 3D‐printed molar replicas, endodontic irrigation, *Enterococcus faecalis* biofilm, Er,Cr:YSGG laser, sonic activation

## Abstract

**Objectives:**

This study compared the efficacy of Sonic (EDDY) and Er,Cr:YSGG (2780 nm) laser activation in eradicating *Enterococcus faecalis* biofilm formed in 3D‐printed molar replicas with two mesial canals and one distal canal.

**Materials and Methods:**

An *in vitro* design was implemented using 20 3D‐printed mandibular molar replicas mimicking the natural canal morphology. Root canals were inoculated with *E. faecalis* and incubated for 21 days to allow the development of a mature biofilm. Three irrigation protocols were tested: Conventional needle irrigation (CNI), EDDY sonic activation (SA), and Er,Cr:YSGG (2780 nm) laser activation (LA). The control group was irrigated with phosphate‐buffered saline (PBS) without activation. Residual bacterial load was quantified through colony‐forming unit (CFU) counts and quantitative PCR (qPCR). Bacterial viability in the apical isthmus was assessed using fluorescence microscopy. A Student's *t*‐test was performed to identify significant differences between CFU/mL values in groups, with significance set at 5% (*p* < 0.05).

**Results:**

CFU counts of *E. faecalis* were significantly lower in the CNI, SA, and LA groups compared to the control (*p* < 0.05). In the SA and LA groups, bacterial counts were reduced to the lower detection limit (< log10 CFU/mL of 1.00), suggesting near‐total bacterial elimination. qPCR and fluorescence microscopy corroborated these results, providing greater differentiation between the outcomes of sonic and laser activations.

**Conclusions:**

Er,Cr:YSGG (2780 nm) laser activation showed superior efficacy in endodontic disinfection by effectively eradicating *E. faecalis* biofilm, including in the challenging isthmus region, representing a promising method for complex root canal anatomies.

## 1 Introduction

Root canal infections represent a significant challenge in endodontics due to the complex microbial biofilms that are formed within the root canal system (Siqueira and Rôças 2022). The biofilm formation and bacterial invasion in canal walls lead to diseases such as apical periodontitis, an inflammatory condition affecting millions worldwide (Olsen 2015). The primary objective of endodontic therapy is to thoroughly eradicate pathogens and prevent recontamination, ensuring long‐term treatment success (Versiani et al. 2016). However, the presence of bacterial biofilms, especially those composed of resilient organisms like *Enterococcus faecalis*, poses a significant challenge to disinfection. Biofilms create a protective environment that enhances bacterial tolerance to conventional antimicrobial treatments (Siqueira and Rôças 2022). Biofilms are structured microbial communities encapsulated within a self‐produced extracellular matrix, which adheres firmly to the surfaces of the root canal walls (Bamford et al. 2023). The phenotypic characteristics of bacteria that reside in these biofilms confer increased resistance to disinfection methods, which, combined with the anatomical intricacies of the root canal system, present a significant challenge to achieving complete bacterial eradication (Stewart 2014). Anatomical complexities and the presence of microbial biofilm structures are the foremost challenges for irrigation (Boutsioukis and Arias‐Moliz 2022). Anatomical features such as isthmuses, lateral canals, and apical ramifications can limit the reach of conventional instruments and irrigants, resulting in unprepared areas along the canal walls. In some cases, 19%–26% of the canal surface may remain untouched, providing a potential reservoir for tissue remnants and bacterial survival (Metzger et al. 2010).

Advanced biofilm management strategies have become a key focus in contemporary endodontic research, incorporating both traditional irrigants and modern irrigation activation techniques designed to enhance the penetration and efficacy of antimicrobial agents (Haapasalo et al. 2014). Traditional irrigation methods predominantly rely on sodium hypochlorite (NaOCl) for its powerful tissue‐dissolving capability and broad‐spectrum antimicrobial properties. However, despite the widespread use of NaOCl, its limited efficacy, particularly in the apical and lateral regions, highlights the need for improved delivery and activation techniques to effectively eradicate persistent biofilms (Haapasalo et al. 2014; de Gregorio et al. 2015).

To overcome the challenges of traditional irrigation, various irrigation activation methods have been developed, including sonic and laser activation techniques. Sonic activation generates acoustic microstreaming through oscillations within the irrigant, which improves biofilm disruption and facilitates disinfectant penetration. Some studies suggest that sonic activation offers advantages over conventional needle irrigation; however, its effectiveness may be limited in certain areas of the root canal (Nusstein 2015; Neuhaus et al. 2016).

Laser‐activated irrigation has emerged as a promising alternative that uses laser‐induced cavitation and photonic energy to achieve superior biofilm removal. Systems like the Er,Cr:YSGG (2780 nm) laser generate rapid energy bursts that create cavitation within the irrigant, resulting in microexplosions that effectively disrupt the biofilm structure (George and Walsh 2010; Koch et al. 2016). Comparative studies have consistently demonstrated the superior efficacy of laser activation in biofilm eradication and enhancing irrigant penetration into difficult‐to‐reach areas, such as isthmuses and lateral canals (Verstraeten et al. 2017).

Despite the potential benefits of agitation and activation methods, few studies have compared their disinfection efficacy in the isthmus region, and such studies traditionally rely on the assessment of colony forming unit (CFU) counts (Josic et al. 2022). The isthmus region is known to harbor a substantial bacterial presence even after chemomechanical preparation, underscoring the need for further investigation into effective disinfection strategies in these challenging anatomical zones (Versiani et al. 2023).

This pilot study aimed to evaluate and compare the effectiveness of sonic and laser activation techniques in endodontic irrigation for removing *E. faecalis* biofilm from the root canals of 3D‐printed tooth models. Additionally, it assessed both activation methods in comparison to conventional manual irrigation using a syringe and needle.

## 2 Materials and Methods

**Figure 1:**
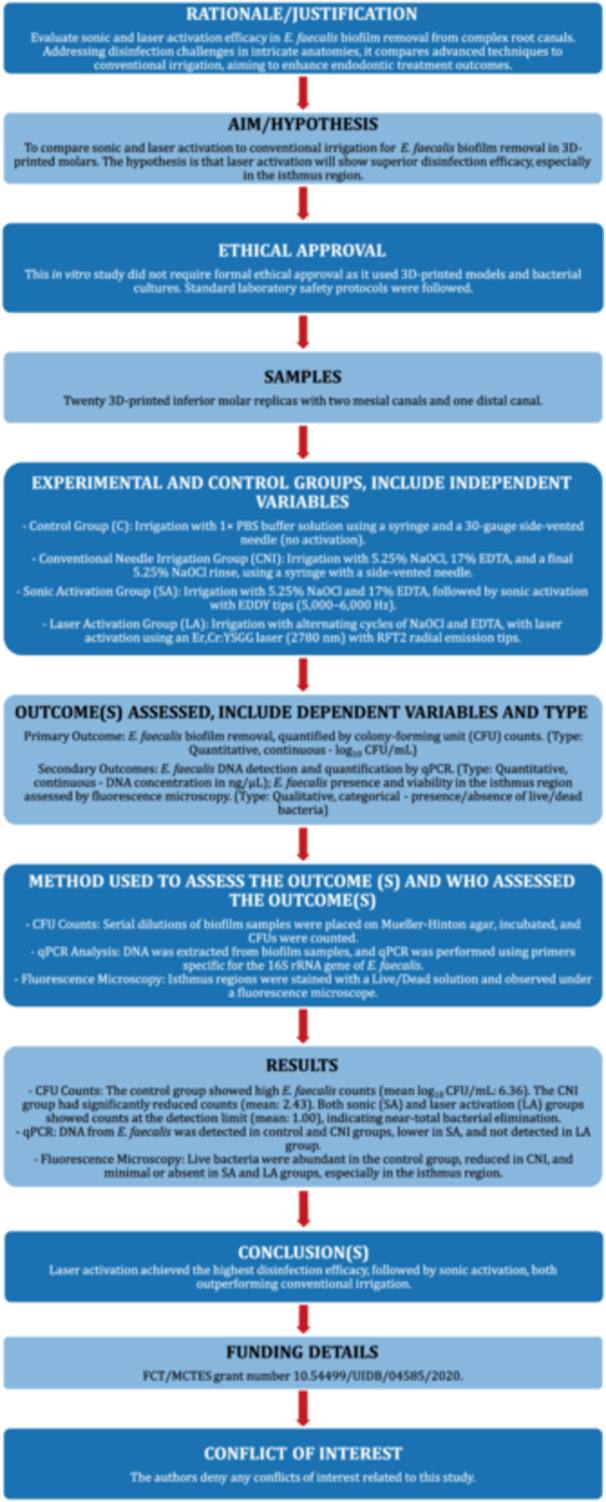
The PRILE 2021 flowchart.

The Preferred Reporting Items for Laboratory studies in Endodontology updated in 2021 (PRILE, http://pride-endodonticguidelines.org/prile) was followed for this laboratory study (Nagendrababu et al. 2021) (Figure 1).

### 2.1 Root Canal Shaping and Preparation in 3D‐Printed Molar Models

**Figure 2:**
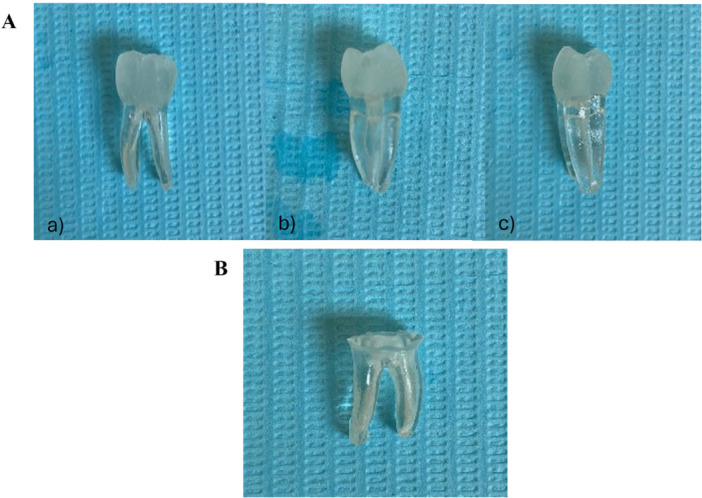
(A) 3D model of a lower molar with two mesial canals and one distal canal (3DsurpreenDente tooth, Portugal), views: (a) buccal, (b) mesial, and (c) distal. (B) 3D model of a lower molar after crown sectioning at the level of the CEJ.

In this study, 25 3D‐printed inferior molar replicas (3DsurpreenDente tooth, Vila Nova de Gaia, Porto, Portugal) were used, each with two roots and three canals (two mesial and one distal) (Figure 2A). The mesial root showed moderate curvature (< 20°) and included an apical isthmus, while the distal root had an oval shape. To standardize sample preparation, the crowns of all replicas were sectioned at the cemento‐enamel junction (CEJ) and eliminated, making all samples with 16 mm (Figure 2B).

Mechanical instrumentation was carried out with BlueShaper rotary files (Zarc4endo, Gijón, Spain) from Z1 to the Z4 file, with a working length of 1 mm short of the total canal length. During instrumentation, each canal was irrigated using a syringe and a 30‐gauge side‐vented needle with 1% NaOCl, applying a back‐and‐forth motion to remove the produced smear layer, followed by a rinse with distilled water to neutralize residual NaOCl. To prevent leakage during biofilm formation, two layers of varnish were applied to the apical surface of each root.

### 2.2 3D‐Printed Models Sterilization Method

Prior to the assay, the 25 3D‐printed replicas were sterilized by filling their canals with 70% ethanol for 20 min, followed by UV light exposure for 2 h (1 h with the canal openings facing down, and another hour with the canal openings facing up). Subsequently, to verify the sterilization efficacy, each model was incubated in BHI broth (Biokar Diagnostics, France) at 37°C for 24 h. The absence of turbidity attested to sterilization efficacy. Two models were contaminated and then discarded; therefore, of the 23 effectively sterilized, we randomly selected 20 for the experimental assays.

### 2.3 Experimental Design and Study Group Allocation

The 20 3D‐printed molar replicas used in this experiment were divided into four groups (*n* = 5 per group), resulting in one experiment with five replicates per group.

Control group (C): Without activation, irrigation with 1 × PBS (phosphate‐buffered saline) using a syringe and a 30‐gauge needle side‐vented.

Conventional needle irrigation group (CNI): Each canal was irrigated with 5.25% NaOCl for 1 min (15 mL), for 1 min, followed by 17% EDTA for 1 min (15 mL), and a final 5.25% NaOCl rinse (15 mL), for 1 min, using a syringe with a side‐vented needle, followed by a PBS rinse for 15 s. A 30‐gauge side‐vented needle was used and positioned 1 mm below the working length. The complete protocol was repeated in each canal. Irrigation was applied with back‐and‐forth movements (Perez et al. 2017).

Sonic activation group (SA): After irrigation with 5.25% NaOCl (12 mL) and 17% EDTA (12 mL), sonic activation was performed with EDDY tips (5000–6000 Hz) with airscaler (KaVo Dental GmbH, Biberach an der Riß, Germany), in three 20‐s cycles, followed by a final PBS rinse for 15 s.

Laser activation group (LA): Laser activation was performed using an Er,Cr:YSGG laser (2780 nm, Waterlase iPlus, Biolase, USA) with RFT2 radial emission tips (200 µm) with the reported settings (1.25 W, 60 µs, 50 Hz, 25 mJ). The irrigation protocol followed the manufacturer's guidelines, with each canal receiving alternating laser activation cycles with both 5.25% NaOCl (12 mL) and EDTA (12 mL). Activation was performed using circular motions at a rate of 2 mm/s, 4 times per canal, for 8 s each canal, with the RFT2 tip positioned 1 mm short of the working length, followed by a final PBS rinse for 15 s.

### 2.4 Inoculum Preparation and Biofilm Formation

An *E. faecalis* ATCC 29212 inoculum was prepared from colonies grown for less than 24 h on Trypticase Soy Agar (TSA) (HiMedia laboratories, India) and suspended in BHI broth. The optical density at 600 nm of the inoculum was adjusted to 0.1, corresponding to approximately 1 × 10^8^ CFU/mL. Then, using a micropipette, the inoculum was added until the entire root canal was filled without overflowing (approximately 20 µL in the distal canal and 10 µL in each mesial canal). The inoculated 20 sterilized 3D‐printed molar replicas were incubated at 37°C in aerobic conditions with 100% humidity for 21 days, with fresh BHI broth added every 48 h.

### 2.5 Biofilm Collection and Quantification by Colony‐Forming Unit (CFU) Counts

Biofilm samples were collected following the Maden et al. (2017) protocol. Briefly, each canal received an ISO #25 sterile paper point inserted into the working length for 60 s. This was followed by scraping with a sterile ISO #25 Hedstroem file for 30 s and another paper point insertion for 60 s. Paper points and file were pooled in 1 mL of PBS (initial suspension) and vortexed for 60 s. Serial dilutions of 10^−1^ to 10^−5^ were prepared, and 100 µL of the initial suspension and each dilution were placed on Mueller‐Hinton agar. CFUs were counted after 24 h of incubation at 37°C, and results were reported as log_10_ CFU/mL.

### 2.6 DNA Extraction and qPCR Analysis

After CFU counting, two out of the initial five suspensions per group were randomly picked for microbial DNA extraction. Those samples were centrifuged at 15,000 × *g* for DNA extraction, which was carried out using the NZYMicrobial gDNA Isolation Kit (NZYtech, Portugal) and according to the manufacturer's instructions. DNA quantification was achieved by qPCR using primers specific for the 16S rRNA gene of *E. faecalis*, as previously described (Takahashi et al. 2011). The PCR reaction (total volume 20 μL) contained 5 μL of microbial genomic DNA, 1×NZYSpeedy qPCR Green Master Mix (NZYtech, Portugal) and 0.5 μL of each primer (forward: 5′‐CCAATCAAATGGCGGCTTCTACG‐3′; reverse: 5′‐GCGATCAGGGAAATGATCGATTCC‐3′).

Amplification was performed using the Rotor‐Gene Q (Qiagen) at 95°C for 10 min, followed by 45 cycles at 95°C for 10 s, 53°C for 10 s, and 72°C for 12 s. To confirm the formation of a single product, a melting curve analysis was performed at 95°C and 55°C for 1 min each, followed by a gradual increase in temperature from 55°C to 95°C at 0.5°C increments every 10 s.

Absolute quantification in each sample was accomplished using the standard curve method. A standard curve was previously constructed as follows. A stock of DNA was obtained from the extraction of a pure culture of *E. faecalis* ATCC 29212, its concentration was measured spectrophotometrically (NanoDrop One/One^C^ Microvolume UV‐Vis Spectrophotometer, Thermo Fisher Scientific, Portugal), and then serially diluted in nuclease‐free water to achieve the following concentrations: 0.1, 0.25, 0.5, 1.0, 2.5, 5.0, 7.5 ng/µL. Each serial dilution was used in separate real‐time reactions, and the corresponding threshold cycle (C_t_) values were determined. The C_t_ values were then plotted against the dilution concentrations on a base‐10 semi‐logarithmic graph, with the data fitted to a straight line. The resulting linear fit demonstrated a correlation coefficient (*R* ^2^) of 0.99.

### 2.7 Fluorescence Microscopy for Bacterial Observation in the Isthmus Region

To visualize the *E. faecalis* biofilm in the isthmus region, two 3D‐printed molar replicas were randomly selected from the five of each experimental group after the biofilm collection, and those replicas were sectioned at the mesial canals in the isthmus region using a 012 diamond‐tapered drill. Then, each isthmus was transferred to a tube containing 500 µL of PBS, vortexed and subjected to ultrasonic treatment for 10 min to disaggregate any remaining biofilm in the isthmus region, centrifuged at 15,000 × *g* for 6 min, after which the supernatant was discarded, and the pellet was resuspended in 100 µL of live/dead solution (BacLight bacterial viability kit, Molecular probes, Thermo Fisher Scientific, USA) and incubated in the dark for 30 min. Following incubation, the samples were centrifuged again under the same conditions and the supernatant was discarded. The isthmus was removed without disturbing the pellet (i.e., the fragment was carefully extracted from the tube with sterile tweezers under direct visual inspection), which was then resuspended in 15 µL of PBS. Finally, 10 µL of the suspension was transferred to a microscope slide, covered with a coverslip, and observed under a fluorescence microscope.

### 2.8 Statistical Analysis

Data for CFU/mL values were analyzed using IBM SPSS Statistics v29 with both descriptive and inferential statistical methods. A Student's *t*‐test was performed to identify significant differences between the C and CNI groups, with statistical significance set at 5% (*p* < 0.05).

## 3 Results

### 3.1 Comparison of CFU Counts of Root Canal‐Adhered *E. faecalis* Across Experimental Groups

**Figure 3:**
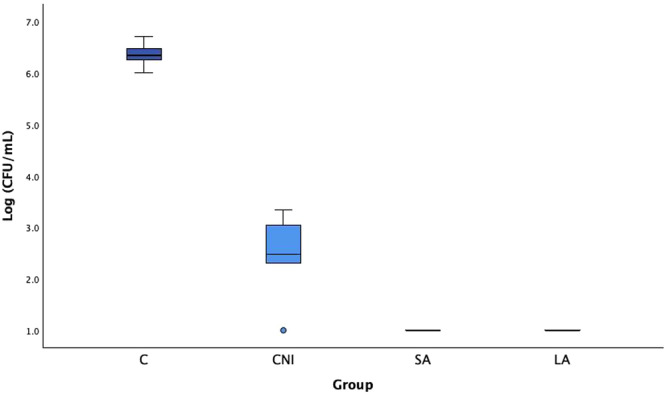
*Enterococcus faecalis* CFU counts (log_10_ CFU/mL) obtained from the no treatment control group (C) and for the treatment groups (conventional needle irrigation (CNI); sonic activation (SA); laser activation (LA)). Mean and standard deviation are presented.

In the C group, a log_10_ CFU/mL of 6.36 (± 0.26) was observed, reflecting a significant bacterial presence due to the absence of any irrigation intervention. In the CNI group, a substantial reduction in CFU/mL count was observed compared to the C group, demonstrating the effectiveness of irrigation without activation (Figure 3).

Although there was some variability in bacterial counts from the five samples of each group, the overall reduction between C and CNI groups was significant (*p* < 0.001). The SA and LA groups exhibited no detectable bacterial count, with Log_10_ CFU/mL values consistently < 1 in all cases (Table 1). The presence of *E. faecalis* was significantly reduced in the CNI, SA, and LA groups compared to the C group. In the SA and LA groups, bacterial counts were reduced to the lower detection limit, log_10_ CFU/mL of 1.00, suggesting near‐total bacterial elimination.

**Table 1:** Descriptive results (log_10_ CFU/mL) for each experimental group.

	Control (C)	CNI	SA	LA
Mean (SD)	6.36 (0.26)*	2.43 (0.90)*	1.000	1.000
Mean (95% CI)	6.04–6.68	1.31–3.56	1.00	1.00
Minimum	6.01	1.00	1.00	1.00
Maximum	6.71	3.34	1.00	1.00

Abbreviations: CI, confidence interval; SD, standard deviation.

* *p* < 0.001 (Student's *t*‐test).

### 3.2 *Enterococcus faecalis* DNA Detection in the Root Canals Across Experimental Groups

Absolute quantification of *E. faecalis* DNA was performed for two samples from each experimental group using the standard curve method (Table 2). Of note, this quantification includes DNA from both live and dead cells. The DNA levels detected in the C and CNI groups were similar, whereas a lower concentration was observed in the SA group, and no DNA was detected in the LA group.

**Table 2:** *Enterococcus faecalis* DNA concentration in the biofilm collected from the root canals of two randomly selected 3D‐printed molar replicas across experimental groups.

Samples from experimental groups	C_t_ values	DNA concentration (ng/µL)
Control‐1	16.57	3.16 × 10^−2^
Control‐2	16.26	3.89 × 10^−2^
CNI‐1	16.01	4.47 × 10^−2^
CNI‐2	18.42	1.02 × 10^−2^
SA‐1	24.28	2.75 × 10^−4^
SA‐2	23.21	5.37 × 10^−4^
LA‐1	Nd	Nd
LA‐2	Nd	Nd

Abbreviation: Nd, non‐detected.

### 3.3 *Enterococcus faecalis* Presence and Viability in the Isthmus Region Across Experimental Groups

**Figure 4:**
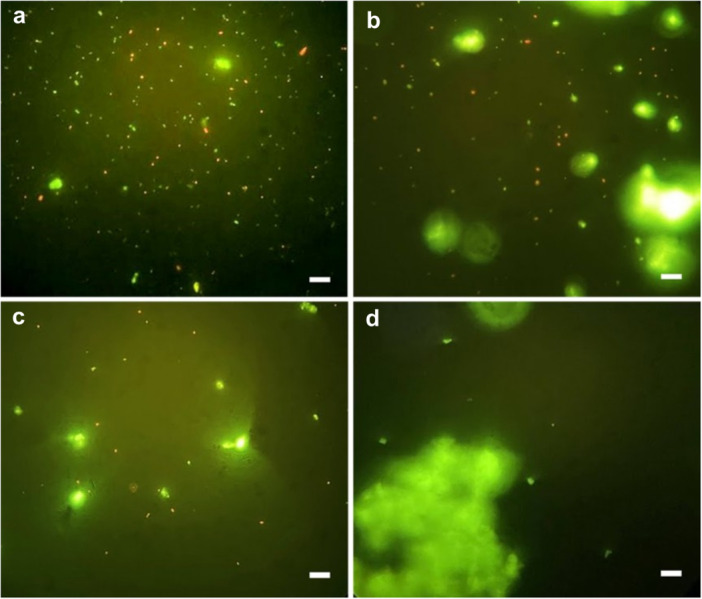
Representative fluorescence microscopy images the residual presence of *E. faecalis* in the isthmus region after live/dead staining; (a) C; (b) CNI; (c) SA; (d) LA groups. The presence of cocci (*E. faecalis*) can be observed, stained green (live) and red (dead). Magnification: 1000 ×. Scale bar: 10 µm.

We further assessed the residual presence of *E. faecalis* in isthmus region in mesial canals, after biofilm collection for CFU quantification, using fluorescence microscopy across all four experimental groups as a complementary qualitative assessment. Representative images, after live/dead staining, were obtained and are shown in Figure 4.

The C group (Figure 4a) showed a markedly higher proportion of green‐stained cells (live bacteria) compared to all other groups, as expected given that no irrigation techniques were applied. In the CNI group (Figure 4b), a reduction in green‐stained cells was observed in comparison to the C group; however, red cells (dead bacteria) are still present. This suggests that this technique may have a less effective flushing capacity compared to the SA and LA groups. The SA group (Figure 4c) also shows a visibly reduced number of both viable and dead *E. faecalis* cells compared to the C and CNI groups, although they still exhibit more stained cells than the LA group. In the LA group (Figure 4d), *E. faecalis* cells were not detected. However, the presence of material debris prevented a definitive assessment of the complete absence of bacteria. These images suggest that laser‐activated irrigation provides greater disinfection efficacy and flushing capacity. The amorphous, larger, green‐stained areas visible in the images correspond to the smear layer, composed of debris from the model molar material, which also fluoresces green. These areas can be distinguished from viable bacteria based on their size and irregular shape.

## 4 Discussion

Considering the critical need for effective cleaning and microbial elimination in the root canal system, this study aimed to evaluate the efficacy of three irrigation techniques in eradicating the *E. faecalis* biofilm, which can be used as an adjunct to mechanical canal debridement.

In this study, results from both quantitative analyses (*E. faecalis* CFU/mL and DNA quantification) and qualitative assessment (fluorescence microscopy) were complementary and reinforced our comparative analysis. The consistency across all three methodologies highlights LA as the most effective approach for reducing bacterial load, with SA also yielding favorable results. Both mechanical activation techniques outperformed conventional needle irrigation in biofilm removal from root canals. Results from both quantitative analyses (*E. faecalis* CFU/mL and DNA quantification) and qualitative assessment (fluorescence microscopy) were complementary and reinforced our comparative analysis. Based on CFU analysis, both SA and LA demonstrated a comparable and near‐complete reduction of viable bacteria, with no detectable differences between these two activation techniques. However, DNA analysis revealed the presence of residual bacterial genetic material only in the SA group, whereas no DNA was detected in the LA group, suggesting a potentially more complete elimination of bacterial remnants when laser activation was applied. The consistency across all three methodologies highlights LA as the most effective approach for reducing bacterial load, with SA also yielding favorable results. Both mechanical activation techniques outperformed conventional needle irrigation in biofilm removal from root canals. These findings are consistent with those reported in previous literature (Josic et al. 2022; Balić et al. 2016; Zhang et al. 2019)

The present study incorporated the complexity of root canal anatomy by using 3D‐printed mandibular molar replicas with mesial canals featuring an isthmus in the apical region. This type of complex anatomy is prevalent in a high proportion of mandibular molars and is often inadequately addressed by mechanical instrumentation alone, placing greater reliance on root canal irrigation for effective disinfection and cleaning (Pereira et al. 2022). Several experimental studies have compared different irrigation techniques and activation of irrigant solutions in straight canals and less complex anatomies (Neuhaus et al. 2016; Urban et al. 2017; Balić et al. 2016). However, a few studies have analyzed and compared these techniques in more challenging ones (Haupt et al. 2020; Zhang et al. 2015).

The bacterium *E. faecalis* was used in this study due to its significant role in endodontic treatment failure and its prevalence in secondary endodontic infections. It is widely accepted that endodontic infections are primarily caused by sessile bacteria that form biofilms. *E. faecalis* is particularly relevant because of its ability to form biofilms, which enhances its resistance and improves its survival conditions (Zhang et al. 2015).

The CFU count is one of the most used methods for evaluating biofilm removal from root canals, as it quantifies viable bacteria on the root canal surface or at various dentin depths (Nusstein 2015; Josic et al. 2022). In this study, the control (C) group served as a reference for bacterial presence in the root canal system without any intervention, and as expected, it exhibited the highest CFU counts. The CNI group showed significantly higher CFU values compared to the SA and LA groups. Although CNI demonstrated some efficacy, the variability among samples suggests that its effectiveness in reducing bacterial load may not be consistent.

*E. faecalis* DNA detection and quantification aligned with the CFU results, with the CNI group exhibiting the highest DNA presence compared to the SA and LA groups. In the LA group, no *E. faecalis* DNA was detected within the detection limit, whereas DNA was still detected in the SA group. The use of qPCR to detect *E. faecalis* DNA in root canals has been reported in the literature, as it enables the identification of bacteria even in low quantities or during the stationary phase, thereby minimizing the risk of false negatives (Balić et al. 2016). While qPCR is a valid method for assessing bacterial presence (Zhang et al. 2019), it does not distinguish between viable and dead cells, which may lead to potential misinterpretations.

The SA group exhibited significantly lower *E. faecalis* CFU counts and DNA levels compared to the control and CNI groups. These consistent findings support the effective flushing capability induced by cavitation waves. The study by Urban et al. (2017) demonstrated that the sonic activation system EDDY had a higher capacity to remove debris from the root canal compared to conventional needle irrigation, results that were corroborated by Haupt et al. (2020) and are also in agreement with the present findings.

Mohmmed et al. (2018) investigated the eradication of *E. faecalis* biofilm in a lateral canal model, comparing irrigation protocols with sonic and ultrasonic agitation using 2.5% NaOCl. Their findings demonstrated that agitation significantly improved canal cleanliness and enhanced irrigant penetration. In the previously mentioned study, the *E. faecalis* biofilm was 10 days old. According to Stojicic et al. (2013) during the first 2 weeks, bacteria in biofilm are sensitive to 1% NaOCl, 2% chlorhexidine, and 0.2/0.4% iodine; however, after 3 weeks, they become highly resistant to these agents. Therefore, in the study by Mohmmed et al. (2018), the observed biofilm eradication may be attributed to its susceptibility to the 2.5% NaOCl concentration. This concentration of NaOCl, combined with agitation techniques, has been reported as sufficient to achieve a significant bacterial reduction. In our study, the biofilm was grown for 3 weeks, making it more resistant to the antimicrobial effects of NaOCl at lower concentrations. Therefore, a 5.25% NaOCl solution was selected.

The LA group was the most effective among the experimental groups, with neither CFUs nor *E. faecalis* DNA detected. This technique seems to promote better penetration of the irrigant solution throughout the canal and superior hydrodynamics and flushing capacity compared to the other techniques, resulting in more efficient biofilm removal (Martins et al. 2022; Betancourt et al. 2019). These results are supported by several studies that compare sonic and ultrasonic techniques with laser activation (Betancourt et al. 2019; Aydin et al. 2020). Therefore, the laser appears to have a superior cavitation effect compared to the sonic system (Martins et al. 2022).

A study by Betancourt et al. demonstrated that Er,Cr:YSGG (2780 nm) laser‐activated irrigation (1 W) enhanced the bactericidal efficiency of 0.5% NaOCl, allowing it to achieve the same level of effectiveness as 5% NaOCl (Betancourt et al. 2019). However, a study by Christo et al. (2016) yielded contradictory results, showing that low‐power Er,Cr:YSGG (2780 nm) laser activation (0.5 W) did not improve the antibacterial effect of NaOCl at low concentrations. This suggests that the low power does not allow a proper cavitation effect, possibly leading to the unintended effect of biostimulation.

Additionally, the microscopic analysis performed to analyze the presence or absence of live or dead residual bacterial cells in the isthmus region revealed that *E. faecalis* was present in greater quantities in the CNI group samples than in the samples from the other two groups (SA, LA). This suggests that the conventional manual irrigation protocol with syringe and needle is less effective, in comparison to the groups that used activation, in promoting fluid dynamics sufficient for the irrigant solution to reach all areas of the root canal, such as the isthmus region.

In the LA group, neither live nor dead cells of *E. faecalis* were detected (Figure 4d). However, the presence of debris from the material itself prevented a definitive confirmation of their complete absence. Nevertheless, microscopic analysis strongly suggested a higher disinfection efficacy and flushing capacity achieved through laser‐activated irrigation. The samples from the SA group also showed a visibly low quantity of both viable and dead *E. faecalis* compared to the control and CNI group samples, but with a higher number of stained cells compared to the LA group samples. The fluorescence microscopy analysis provided a visual confirmation of the impact of the treatments, reinforcing the importance of activation as a key factor in disinfection and cleaning of teeth with more complex anatomies, such as isthmus areas.

Sonic activation with flexible EDDY tips may provide an advantage over the conventional 30G needle by enabling better access to the apical portion of the canal, even in sharply curved root canals, while maintaining oscillation despite contact with canal walls due to their non‐cutting tip (Urban et al. 2017). However, further studies are required to evaluate EDDY's cleaning efficacy in complex canal configurations.

The Er,Cr:YSGG (2780 nm) laser with radial firing tips (RFTs) enhances endodontic irrigation by leveraging cavitation effects to improve fluid dynamics. A study by Martins et al. (2022) demonstrates that, unlike conventional methods, the Er,Cr:YSGG (2780 nm) laser generates high‐speed vapor bubbles, inducing secondary cavitation and negative apical pressure, which enhances fluid penetration. The radial energy dispersion of RFTs directs laser energy laterally along the canal walls, minimizing the risk of apical extrusion while maximizing cleaning efficiency. High‐speed imaging confirms that cavitation bubbles persist beyond the laser pulse, further amplifying debris removal. Given these advantages, the Er,Cr:YSGG (2780 nm) laser with RFTs offers a safer and more effective approach to root canal disinfection compared to traditional irrigation techniques. Some studies on laser activation have explored various power settings and radiation energy levels, positioning the tip up to 5 mm from the apex or even within the pulp chamber, while achieving comparable bactericidal effects (Koch et al. 2016; Golob et al. 2017). This approach enhances irrigation effectiveness in curved and complex root canal anatomies.

Despite the growing body of research comparing sonic and laser‐activated irrigation systems, a standardized methodology for evaluating their effectiveness remains elusive (Boutsioukis et al. 2022). Future studies should prioritize harmonizing experimental conditions, including biofilm incubation time, irrigation protocols, and standardized evaluation criteria, to facilitate direct comparisons across studies. A critical limitation in current research is the challenge of fully retrieving biofilm from root canals due to their anatomical complexity. This complexity often leads to inconsistencies in microbial quantification, impacting the reliability of antimicrobial efficacy assessments (Boutsioukis et al. 2022).

To overcome these limitations, combining complementary techniques such as fluorescence microscopy and quantitative polymerase chain reaction (qPCR) has been proposed to improve the accuracy of disinfection efficacy assessments (Boutsioukis et al. 2022). However, standard qPCR methods may overestimate bacterial viability by detecting DNA from both live and dead bacteria. To overcome this issue, future investigations should consider incorporating propidium monoazide treatment before qPCR analysis, as suggested by Salam et al. (2014) to selectively exclude DNA from membrane‐compromised cells. This approach would provide a more accurate differentiation between viable and non‐viable bacteria, leading to a more representative evaluation of bacterial eradication across different irrigation and activation protocols.

Furthermore, optimizing methodologies for assessing irrigant penetration and apical extrusion is essential. Transparent root canal models and numerical simulations have been recommended as effective tools for studying fluid dynamics within the canal system, while micro‐computed tomography (micro‐CT) allows for detailed visualization of accumulated dentine debris and its removal following irrigation (Boutsioukis et al. 2022). Future research should incorporate these advanced techniques to enhance the clinical relevance of in vitro findings and ensure their effective translation into practice.

All 3D‐printed mandibular molar replicas used in this study were fabricated from transparent synthetic materials, whose surface properties and composition differ from natural dentin. Unlike these standardized 3D replicas, natural teeth exhibit distinct structural and biological characteristics, which may influence bacterial adhesion and biofilm formation (Mohmmed et al. 2018). While laboratory studies using ex vivo extracted teeth may offer greater clinical relevance for assessing the antimicrobial efficacy of irrigation techniques, the in vitro 3D model developed in this study presents key advantages. The primary strength of this model is its high level of standardization, which eliminates confounding variables such as root canal anatomy, dentin composition, and preparation inconsistencies typically associated with extracted teeth. Additionally, it overcomes ethical concerns and the logistical challenges associated with obtaining ex vivo natural teeth that meet specific research criteria. The 3D‐printed replicas were produced in series with identical anatomical characteristics, ensuring reproducibility and enhancing the reliability of experimental conditions.

A potential limitation of this approach is that biofilm adhesion to synthetic materials may differ from its interaction with dentin. However, in the control group, *E. faecalis* biofilm successfully formed on the 3D‐printed replicas, demonstrating that the model effectively supports bacterial growth and development. Furthermore, the low standard deviation in CFU counts across the five replicates suggests a high degree of consistency in biofilm formation and quantification among the tested molar replicas. Notably, previous studies analyzing *E. faecalis* biofilm formation on natural teeth have reported similar values to those obtained in this pilot study using 3D‐printed replicas (Bago Jurič et al. 2016; Betancourt et al. 2020). This supports the suitability of 3D‐printed teeth as a clinically relevant alternative for research, enabling appropriate sample sizes while eliminating anatomical variability.

Laser activation with Er,Cr:YSGG (2780 nm) lasers and RFTs has demonstrated significant potential in biofilm removal and endodontic disinfection by enhancing biofilm disruption and fluid dynamics. However, further research is required to evaluate its antimicrobial efficacy when used without irrigating solutions and to determine the impact of key parameters such as laser power, pulse duration, and tip insertion depth. Furthermore, the clinical implications of laser activation within curved root canals, along with its potential effects on postoperative healing and pain, warrant further investigation to validate the safety and therapeutic efficacy of this technique. Given its promising results, laser‐activated irrigation may offer a more effective alternative to conventional irrigation, particularly in anatomically complex canals, but further studies are essential to optimize protocols and validate its long‐term clinical benefits.

## Author Contributions

**João Albernaz Neves** and **Alejandro R. Pérez:** conceptualization. **Margarida Macedo** and **Lucinda J. Bessa:** methodology. **Margarida Macedo, João Albernaz Neves, Luís Proença**, and **Lucinda J. Bessa:** data analysis. **Margarida Macedo** and **Lucinda J. Bessa:** investigation. **Margarida Macedo** and **Lucinda J. Bessa:** writing – original draft preparation. **João Albernaz Neves, Alejandro R. Pérez, Luís Proença**, and **Lucinda J. Bessa:** writing – review and editing. **Margarida Macedo** and **Luís Proença:** visualization. All authors have read and agreed to the published version of the manuscript.

## Conflicts of Interest

The authors declare no conflicts of interest.

## Supporting information

cre2.20250448‐File008.

## Data Availability

The data that support the findings of this study are available from the corresponding author upon reasonable request.

## References

[cre270279-bib-0001] Aydin, S. A. , T. Taşdemir , C. K. Buruk , and D. Çelik . 2020. “Efficacy of Erbium, Chromium‐Doped Yttrium, Scandium, Gallium and Garnet Laser‐Activated Irrigation Compared With Passive Ultrasonic Irrigation, Conventional Irrigation, and Photodynamic Therapy Against *Enterococcus faecalis* .” Journal of Contemporary Dental Practice 21, no. 1: 11–16. 32381794

[cre270279-bib-0002] Bago Jurič, I. , V. Plečko , I. Anić , et al. 2016. “Antimicrobial Efficacy of Photodynamic Therapy, Nd:YAG Laser and QMiX Solution Against *Enterococcus faecalis* Biofilm.” Photodiagnosis and Photodynamic Therapy 13: 238–243. 10.1016/j.pdpdt.2015.07.176. 26232719

[cre270279-bib-0003] Balić, M. , R. Lucić , K. Mehadžić , et al. 2016. “The Efficacy of Photon‐Initiated Photoacoustic Streaming and Sonic‐Activated Irrigation Combined With QMiX Solution or Sodium Hypochlorite Against Intracanal *E. faecalis* Biofilm.” Lasers in Medical Science 31, no. 2: 335–342. 10.1007/s10103-015-1864-9. 26754179

[cre270279-bib-0004] Bamford, N. C. , C. E. MacPhee , and N. R. Stanley‐Wall . 2023. “Microbial Primer: An Introduction to Biofilms—What They Are, Why They Form and Their Impact on Built and Natural Environments.” Microbiology 169, no. 8: 001338. 10.1099/mic.0.001338. 37526065 PMC7615007

[cre270279-bib-0005] Betancourt, P. , A. Merlos , J. M. Sierra , J. Arnabat‐Dominguez , and M. Viñas . 2020. “Er,Cr:YSGG Laser‐Activated Irrigation and Passive Ultrasonic Irrigation: Comparison of Two Strategies for Root Canal Disinfection.” Photobiomodulation, Photomedicine, and Laser Surgery 38, no. 2: 91–97. 10.1089/photob.2019.4645. 31397611

[cre270279-bib-0006] Betancourt, P. , A. Merlos , J. M. Sierra , O. Camps‐Font , J. Arnabat‐Dominguez , and M. Viñas . 2019. “Effectiveness of Low Concentration of Sodium Hypochlorite Activated by Er,Cr:YSGG Laser Against *Enterococcus faecalis* Biofilm.” Lasers in Medical Science 34, no. 2: 247–254. 10.1007/s10103-018-2578-6. 29980946

[cre270279-bib-0007] Boutsioukis, C. , and M. T. Arias‐Moliz . 2022. “Present Status and Future Directions—Irrigants and Irrigation Methods.” International Endodontic Journal 55, no. 3: 588–612. 10.1111/iej.13739. 35338652 PMC9321999

[cre270279-bib-0008] Boutsioukis, C. , M. T. Arias‐Moliz , and L. E. Chávez de Paz . 2022. “A Critical Analysis of Research Methods and Experimental Models to Study Irrigants and Irrigation Systems.” International Endodontic Journal 55, no. S2: S295–S329. 10.1111/iej.13710. PMC9314845 35171506

[cre270279-bib-0009] Christo, J. E. , P. S. Zilm , T. Sullivan , and P. R. Cathro . 2016. “Efficacy of Low Concentrations of Sodium Hypochlorite and Low‐Powered Er,Cr:YSGG Laser Activated Irrigation Against an *Enterococcus faecalis* Biofilm.” International Endodontic Journal 49, no. 3: 279–286. 10.1111/iej.12447. 25772335

[cre270279-bib-0010] George, R. , and L. J. Walsh . 2010. “Thermal Effects From Modified Endodontic Laser Tips Used in the Apical Third of Root Canals With Erbium‐Doped Yttrium Aluminium Garnet and Erbium, Chromium‐Doped Yttrium Scandium Gallium Garnet Lasers.” Photomedicine and Laser Surgery 28, no. 2: 161–165. 10.1089/pho.2008.2423. 20201662

[cre270279-bib-0011] Golob, B. S. , G. Olivi , M. Vrabec , R. El Feghali , S. Parker , and S. Benedicenti . 2017. “Efficacy of Photon‐Induced Photoacoustic Streaming in the Reduction of *Enterococcus faecalis* Within the Root Canal: Different Settings and Different Sodium Hypochlorite Concentrations.” Journal of Endodontics 43, no. 10: 1730–1735. 10.1016/j.joen.2017.05.019. 28756961

[cre270279-bib-0012] de Gregorio, C. , A. Arias , N. Navarrete , R. Cisneros , and N. Cohenca . 2015. “Differences in Disinfection Protocols for Root Canal Treatments Between General Dentists and Endodontists.” Journal of the American Dental Association 146, no. 7: 536–543. 10.1016/j.adaj.2015.01.027. 26113101

[cre270279-bib-0013] Haapasalo, M. , Y. Shen , Z. Wang , and Y. Gao . 2014. “Irrigation in Endodontics.” British Dental Journal 216, no. 6: 299–303. 10.1038/sj.bdj.2014.204. 24651335

[cre270279-bib-0014] Haupt, F. , M. Meinel , A. Gunawardana , and M. Hülsmann . 2020. “Effectiveness of Different Activated Irrigation Techniques on Debris and Smear Layer Removal From Curved Root Canals: A SEM Evaluation.” Australian Endodontic Journal 46, no. 1: 40–46. 10.1111/aej.12342. 30907051

[cre270279-bib-0015] Josic, U. , C. Mazzitelli , T. Maravic , A. Fidler , L. Breschi , and A. Mazzoni . 2022. “Biofilm in Endodontics: In Vitro Cultivation Possibilities, Sonic‐, Ultrasonic‐ and Laser‐Assisted Removal Techniques and Evaluation of the Cleaning Efficacy.” Polymers 14, no. 7: 1334. 10.3390/polym14071334. 35406207 PMC9003475

[cre270279-bib-0016] Koch, J. D. , D. E. Jaramillo , E. DiVito , and O. A. Peters . 2016. “Irrigant Flow During Photon‐Induced Photoacoustic Streaming (PIPS) Using Particle Image Velocimetry (PIV).” Clinical Oral Investigations 20, no. 2: 381–386. 10.1007/s00784-015-1562-9. 26303646

[cre270279-bib-0017] Maden, M. , I. F. Ertuğrul , E. O. Orhan , et al. 2017. “Enhancing Antibacterial Effect of Sodium Hypochlorite by Low Electric Current‐Assisted Sonic Agitation.” PLoS One 12, no. 8: e0183895. 10.1371/journal.pone.0183895. 28854274 PMC5576683

[cre270279-bib-0018] Martins, M. R. , R. De Moor , N. Gutknecht , and R. Franzen . 2022. “Endodontic Impact of Cavitation and Bubble Formation Induced by 2780‐nm Er,Cr:YSGG Laser Using Radial Firing Tips on Simulated Root Canals.” Lasers in Dental Science 6: 195–204. 10.1007/s41547-022-00160-3.

[cre270279-bib-0019] Metzger, Z. , R. Zary , R. Cohen , E. Teperovich , and F. Paqué . 2010. “The Quality of Root Canal Preparation and Root Canal Obturation in Canals Treated With Rotary Versus Self‐Adjusting Files: A Three‐Dimensional Micro‐Computed Tomographic Study.” Journal of Endodontics 36, no. 9: 1569–1573. 10.1016/j.joen.2010.06.003. 20728729

[cre270279-bib-0020] Mohmmed, S. A. , M. E. Vianna , M. R. Penny , S. T. Hilton , N. J. Mordan , and J. C. Knowles . 2018. “Investigations Into In Situ *Enterococcus faecalis* Biofilm Removal by Passive and Active Sodium Hypochlorite Irrigation Delivered Into the Lateral Canal of a Simulated Root Canal Model.” International Endodontic Journal 51, no. 6: 649–662. 10.1111/iej.12880. 29178348

[cre270279-bib-0021] Nagendrababu, V. , P. E. Murray , R. Ordinola‐Zapata , et al. 2021. “PRILE 2021 Guidelines for Reporting Laboratory Studies in Endodontology: A Consensus‐Based Development.” International Endodontic Journal 54, no. 9: 1482–1490. 10.1111/iej.13542. 33938010

[cre270279-bib-0022] Neuhaus, K. W. , M. Liebi , S. Stauffacher , S. Eick , and A. Lussi . 2016. “Antibacterial Efficacy of a New Sonic Irrigation Device for Root Canal Disinfection.” Journal of Endodontics 42, no. 12: 1799–1803. 10.1016/j.joen.2016.08.024. 27780580

[cre270279-bib-0023] Nusstein, J. M. 2015. “Sonic and Ultrasonic Irrigation.” In Endodontic Irrigation, edited by B. Basrani . Springer. 10.1007/978-3-319-16456-4_10.

[cre270279-bib-0024] Olsen, I. 2015. “Biofilm‐Specific Antibiotic Tolerance and Resistance.” European Journal of Clinical Microbiology & Infectious Diseases 34, no. 5: 877–886. 10.1007/s10096-015-2323-z. 25630538

[cre270279-bib-0025] Pereira, M. R. , P. Pascoal‐Faria , I. Vasconcelos , N. Alves , and A. Ginjeira . 2022. “Endodontics Irrigation: A Computational Fluid Dynamics Approach.” AIP Conference Proceedings 2425, no. 1: 220002. 10.1063/5.0085908.

[cre270279-bib-0026] Perez, R. , A. A. Neves , F. G. Belladonna , et al. 2017. “Impact of Needle Insertion Depth on the Removal of Hard‐Tissue Debris.” International Endodontic Journal 50, no. 6: 560–568. 10.1111/iej.12648. 27061910

[cre270279-bib-0027] Salam, K. W. , M. El‐Fadel , E. K. Barbour , and P. E. Saikaly . 2014. “A Propidium Monoazide‐Quantitative PCR Method for the Detection and Quantification of Viable *Enterococcus faecalis* in Large‐Volume Samples of Marine Waters.” Applied Microbiology and Biotechnology 98, no. 20: 8707–8718. 10.1007/s00253-014-6023-x. 25149448

[cre270279-bib-0028] Siqueira, Jr., J. F. , and I. N. Rôças . 2022. “Present Status and Future Directions: Microbiology of Endodontic Infections.” International Endodontic Journal 55, no. 3: 512–530. 10.1111/iej.13677. 34958494

[cre270279-bib-0029] Stewart, P. S. 2014. “Biophysics of Biofilm Infection.” Pathogens and Disease 70, no. 3: 212–218. 10.1111/2049-632X.12118. 24376149 PMC3984611

[cre270279-bib-0030] Stojicic, S. , Y. Shen , and M. Haapasalo . 2013. “Effect of the Source of Biofilm Bacteria, Level of Biofilm Maturation, and Type of Disinfecting Agent on the Susceptibility of Biofilm Bacteria to Antibacterial Agents.” Journal of Endodontics 39, no. 4: 473–477. 10.1016/j.joen.2012.11.024. 23522539

[cre270279-bib-0037] Takahashi, Y. , A. Yoshida , M. Nagayoshi , et al. 2011. “Enumeration of Viable Enterococcus faecalis, a Predominant Apical Periodontitis Pathogen, Using Propidium Monoazide and Quantitative Real‐Time Polymerase Chain Reaction.” Microbiology and Immunology 55, no. 12: 889–892. 10.1111/j.1348-0421.2011.00390.x. 22003816

[cre270279-bib-0031] Urban, K. , D. Donnermeyer , E. Schäfer , and S. Bürklein . 2017. “Canal Cleanliness Using Different Irrigation Activation Systems: A SEM Evaluation.” Clinical Oral Investigations 21, no. 9: 2681–2687. 10.1007/s00784-017-2070-x. 28185091

[cre270279-bib-0032] Versiani, M. A. , F. R. F. Alves , C. V. Andrade‐Junior , et al. 2016. “Micro‐CT Evaluation of the Efficacy of Hard‐Tissue Removal From the Root Canal and Isthmus Area by Positive and Negative Pressure Irrigation Systems.” International Endodontic Journal 49, no. 11: 1079–1087. 10.1111/iej.12559. 26459183

[cre270279-bib-0033] Versiani, M. A. , J. Martins , and R. Ordinola‐Zapata . 2023. “Anatomical Complexities Affecting Root Canal Preparation: A Narrative Review.” Australian Dental Journal 68, no. S1: S5. 10.1111/adj.12992. 37984802

[cre270279-bib-0034] Verstraeten, J. , W. Jacquet , R. J. G. De Moor , and M. A. Meire . 2017. “Hard Tissue Debris Removal From the Mesial Root Canal System of Mandibular Molars With Ultrasonically and Laser‐Activated Irrigation: A Micro‐Computed Tomography Study.” Lasers in Medical Science 32, no. 9: 1965–1970. 10.1007/s10103-017-2297-4. 28782092

[cre270279-bib-0035] Zhang, C. , J. Du , and Z. Peng . 2015. “Correlation Between *Enterococcus faecalis* and Persistent Intraradicular Infection Compared With Primary Intraradicular Infection: A Systematic Review.” Journal of Endodontics 41, no. 8: 1207–1213. 10.1016/j.joen.2015.04.008. 26015157

[cre270279-bib-0036] Zhang, D. , Y. Shen , C. de la Fuente‐Núñez , and M. Haapasalo . 2019. “In Vitro Evaluation by Quantitative Real‐Time PCR and Culturing of the Effectiveness of Disinfection of Multispecies Biofilms in Root Canals by Two Irrigation Systems.” Clinical Oral Investigations 23, no. 2: 913–920. 10.1007/s00784-018-2515-x. 29948281

